# Testing the Sentinel Method: Live and Artificial Prey Display Contrasting Patterns of Predation Across an Urban Gradient

**DOI:** 10.1002/ece3.72675

**Published:** 2025-12-17

**Authors:** Yu Zeng, Haolin Yang, Yiheng Pan, Yuxuan Li, Dohee Kim, Haokun Wang, Jing Feng, Yuechen Huang, Yingjie Yin, Hanqing Zhao, Yuyang Wu, Craig R. A. Barnett, Catherine L. Parr, Samantha C. Patrick, Yi Zou, Emilio Pagani‐Núñez

**Affiliations:** ^1^ Department of Health and Environmental Sciences, School of Science Xi'an Jiaotong‐Liverpool University Suzhou China; ^2^ School of Advanced Technology Xi'an Jiaotong‐Liverpool University Suzhou China; ^3^ Department of Biosciences and Bioinformatics Xi'an Jiaotong‐Liverpool University Suzhou China; ^4^ Department of Zoology, Graduate School of Science Kyoto University Kyoto Japan; ^5^ Department of Earth, Ocean and Ecological Sciences, School of Environmental Science University of Liverpool Liverpool UK; ^6^ Department of Zoology & Entomology University of Pretoria Pretoria South Africa; ^7^ School of Animal, Plant and Environmental Sciences University of the Witwatersrand Wits South Africa; ^8^ Centre for Conservation and Restoration Science Edinburgh Napier University Edinburgh UK; ^9^ School of Applied Sciences Edinburgh Napier University Edinburgh UK

**Keywords:** ecosystem services, plasticine prey, predation, sentinel method, urbanization

## Abstract

Assessing changes in the intensity of biotic interactions across environmental gradients is a central issue in ecology. The sentinel method has been widely adopted to study predator–prey interactions by establishing patches of prey under different conditions that predators can attack. Sentinels, proxies for prey, are frequently worm‐shaped prey resembling caterpillars and are commonly used to assess predation by arthropod‐feeding predators, with predation measured as the rate of disappearance or evidence of predation after a certain period of exposure. While it has been suggested that artificial sentinel prey might produce divergent results from live prey, previous studies showed mixed results in the difference between these two prey types. Results are likely to vary with context, and the assessment of different prey types along urban gradients is still lacking. Here, we performed an experiment at 10 sites across a natural‐to‐urban gradient in Suzhou (East China) combining live prey and artificial prey to determine differences in predation intensity between these prey types. We released 2575 artificial prey and 3825 live prey, either separately (artificial or live prey alone) or combined, in a randomized sequence. We found a positive relationship between our index of predation and the level of urbanization using both types of prey. However, the predation rate using artificial prey was lower than with live prey and showed a different pattern with urbanization. The predation rate using live prey was higher for avian predators and lower for insect predators with increasing urbanization. Our results show that artificial and live prey can produce divergent estimates of predation intensity. Thus, while artificial prey may be used as a rapid‐screening tool, live prey could be favored in comprehensive studies to assess this fundamental ecosystem service.

## Graphical abstract

We examined predation intensity on live and artificial prey across a natural‐to‐urban gradient in Suzhou, China. Our findings revealed that artificial prey underestimated predation rates compared to live prey, with different patterns observed between avian and insect predators along the urbanization gradient. While artificial prey offers advantages for predator identification, live prey is more accurate for comprehensive assessments, emphasizing the need to consider sentinel prey type in ecological studies of predation.

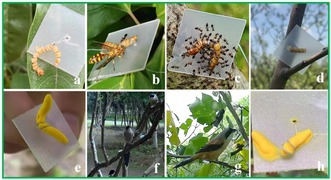

## 1 Introduction

Predation, the ecological process by which a predator captures and immobilizes and/or kills a prey before eating it, is a major ecological and evolutionary force (Taylor 2013) and a central ecosystem service (Gilbert et al. 2021). However, predation is difficult to study as it often happens quickly and leaves no trace. This is particularly apparent for arthropods, which act as both predators and prey, given their small size and high speed of movement. Consequently, a common approach to quantify predation involves establishing patches of arthropod prey, which are usually referred to as sentinels (Lövei and Ferrante 2017). Sentinel prey are set in the environment over a fixed time and area, while predation is recorded as the disappearance of the sentinel or the number of marks left on the sentinel by different predators (Birkhofer et al. 2017; Low et al. 2014). There are two main approaches to implementing the sentinel method, using either live prey or alternatives such as dead or artificial prey. Live sentinel prey often include various types of arthropods, primarily insects; dead prey are usually similar in form but lack movement or chemical cues; while artificial prey typically resemble worms and generally are made of plasticine (Lövei and Ferrante 2017). These two approaches have produced contrasting results, with live prey producing higher predation rates than dead or plasticine prey on average, and with predators from different taxa responding differently to these prey types, for example, birds prefer live prey whereas grasshoppers prefer dead prey (Lövei and Ferrante 2017; Zou et al. 2017).

Most field studies assessing predation along environmental gradients, such as urbanization, altitude, or latitude gradients, either use live prey or artificial models (Lövei and Ferrante 2017). The differences between them are likely to introduce biases in our estimates of predation and more generally in how biotic interactions change across environmental gradients (Zvereva and Kozlov 2021), particularly when comparing among studies. For instance, studies testing the hypothesis that predator–prey interactions increase nearer the equator have produced contrasting results. Results from a large‐scale experiment using artificial prey suggested that predation intensity increases toward the equator and that this increase is mostly due to arthropod, rather than vertebrate, predation, with ants acting as main arthropod predators globally and particularly in tropical regions (Roslin et al. 2017). However, a recent study found that this poleward decrease in predation was only found using plasticine prey, with this relationship being non‐significant for live prey (Zvereva et al. 2024). In addition, a comparison of the performance of these two prey types in boreal forests showed that ant predators prefer live rather than artificial prey, while the latter were only attacked by avian predators (Zvereva and Kozlov 2023). Moreover, recent studies using insect herbivore damage on plants as an indicator of predator control on insect populations have shown that vertebrates are the main predators of arthropods in a tropical area (Houska et al. 2023; Sam et al. 2023). There are also technical issues related to the use of plasticine prey since plasticine can melt in high temperatures and freeze in cold conditions, which strongly affects predation rate estimations (Muchula et al. 2019). Therefore, further research is needed to assess the performance of artificial and live prey across diverse environmental gradients.

Urban gradients provide an ideal scenario to assess how the use of live or artificial prey affects our estimates of arthropod predation intensity and, consequently, the intensity of biotic interactions. A meta‐analysis has suggested predation declines with increasing urbanization, potentially due to reduced predator diversity, lower prey abundance, and the disruption of trophic interactions in more urbanized areas, but it only included one study using the sentinel method (Eötvös et al. 2018). Studies using artificial prey in urban settings have also produced mixed results, with some studies reporting decreasing predation with increasing urbanization (Lidasan et al. 2023; Pena et al. 2021), while others found the opposite pattern (Cupitra‐Rodríguez et al. 2023; Kozlov et al. 2017). Additional research has revealed contrasting patterns of predation in response to human pressure across latitudes (Alonso‐Crespo and Hernández‐Agüero 2023), and across predator taxa (Ferrante et al. 2014). Although the use of artificial prey is widespread, it may introduce systematic biases in estimates of predation intensity. For instance, urbanization may alter predator–prey interactions by modifying predator abundance and composition, such as increasing the abundance of generalist bird predators and reducing insect predator diversity and activity (Shochat et al. 2006; Fenoglio et al. 2020). However, there have been no attempts to assess the performance of live and artificial prey across urban gradients while also distinguishing between bird and insect predators. Ascertaining predation intensity across urban gradients with accuracy is especially important due to the negative effects of urbanization on arthropod population control (Korányi et al. 2022).

Here, we conducted a test of the sentinel method across an urban gradient in Suzhou (eastern China) using both artificial prey and live sentinel prey. Our two main objectives in this study were to: (1) assess how the use of live and artificial prey might affect estimates of predation intensity across urban gradients and (2) investigate how different predators (birds and insects) respond to live and artificial prey. According to the predatory‐relaxation hypothesis, predation pressure is expected to decline in more urbanized environments due to reduced predator diversity and abundance, which is an idea supported by meta‐analytic and experimental findings (Eötvös et al. 2018; Gering and Blair 1999). However, previous studies using artificial prey have produced inconsistent results (e.g., Kozlov et al. 2017; Pena et al. 2021). Our first objective is therefore important, as it evaluates whether using artificial versus live prey affects the detection of urbanization‐driven patterns in predation. The second objective is important because there is still much debate regarding the importance of different taxa as predators of insects. For example, a previous study on a boreal forest of northern Europe showed that ant predators consumed most live prey and birds only attacked artificial prey (Zvereva and Kozlov 2023). However, studies in temperate and subtropical urban sites in East Asia and natural sites in Papua New Guinea have reported intense bird predation on live (He et al. 2022; Yamazaki et al. 2020) and artificial (Sam et al. 2023) prey.

## 2 Materials and Method

### 2.1 Study Area

**FIGURE 1:**
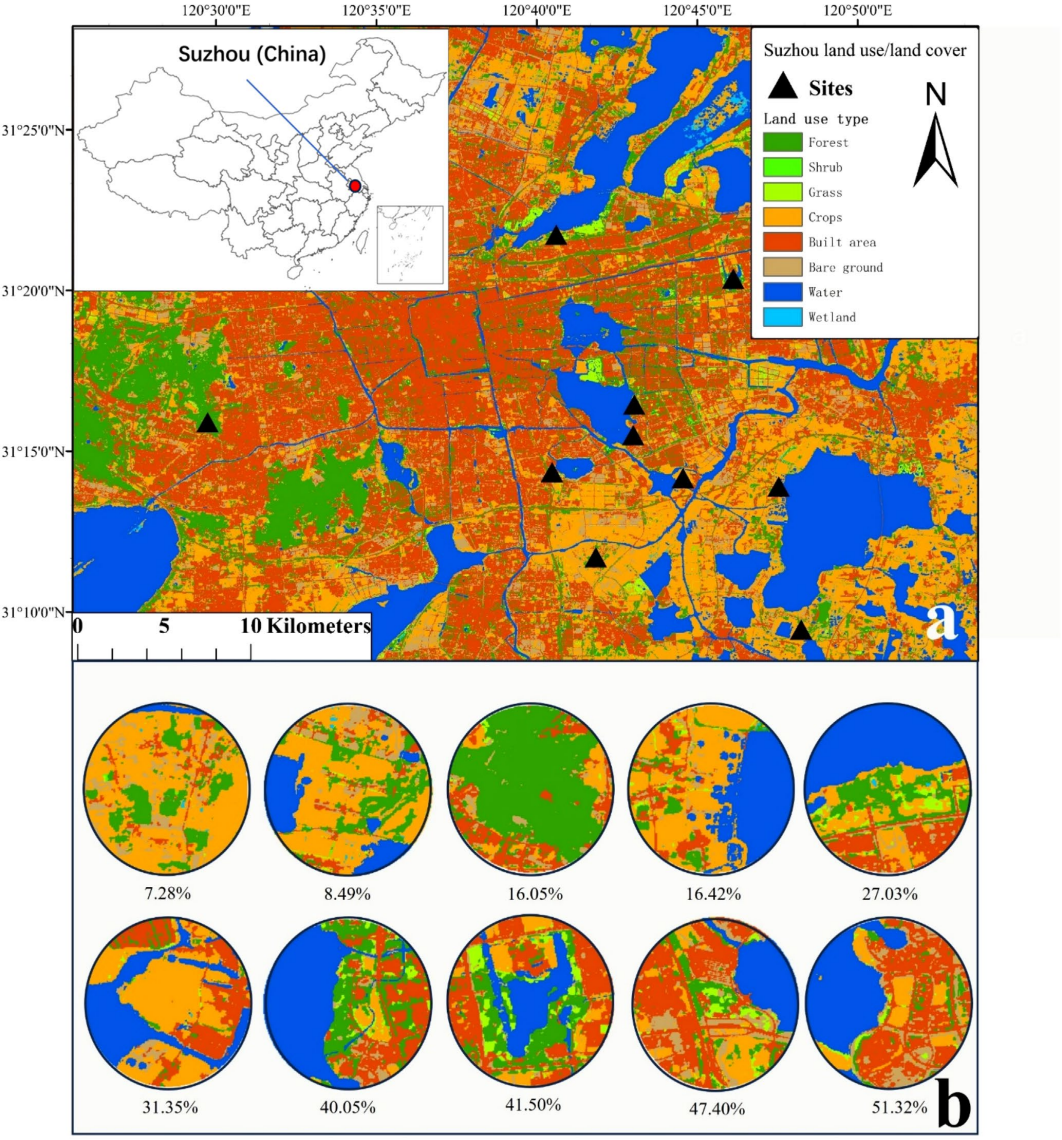
Study sites distribution in Suzhou (China) and urbanization rate quantification. Ten sites (black triangles) with different urbanization rates were selected (a). Land use type was identified as forest, shrub, grass, crops, built area, bare ground, water, and wetland. The urbanization rate for each site was calculated by analyzing land use data within a standard radius of 1 km (b). We used the built‐up area and divided the total land area after excluding water bodies.

This experiment was conducted in Suzhou (30°47′–32°02′ N, 119°55′–121°20′), Jiangsu province, China (Figure 1a). Suzhou is situated within the humid monsoon climate zone of China's subtropical region, with humid and rainy summers and dry, cold winters. Suzhou's combination of urban development and preserved natural habitat makes it an ideal location for studying the impact of urbanization on wildlife (Wang et al. 2015). This experiment was conducted at 10 sites following an urbanization gradient from natural reserves and agricultural farmlands to urban parks from December 2020 to August 2021. We visited each site twice, once during the winter and another during the summer. We used ArcGIS (10.8) to quantify the urbanization rate using 10‐m spatial resolution land use/land cover (LULC) data of Suzhou City political boundary from Geographical Information Monitoring Cloud Platform (http://www.dsac.cn) and Google Earth (https://earth.google.com/). The urbanization rate was quantified using the percentage of built area (Guo et al. 2016). Each site was extracted from the original LULC map within a standard circle with a radius of 1 km around the central point. The urbanization rate of each site was quantified using built area divided by the whole area, from which the water area was excluded since there is a large portion of water area in some sites which could be a bias. The urbanization rates of the 10 sites were evenly distributed from 7.28% to 51.32% built‐up area (Figure 1b). For detailed values and land cover data of each site, see Table S1.

### 2.2 Sentinel Prey Preparation

To compare the performance of quantifying predation intensity by live and artificial prey, we used mealworms ( *Tenebrio molitor* ) as live prey and yellow plasticine molded into worm shapes (mimicking mealworms' coloration) as artificial prey. Both live mealworms and plasticine prey were individually attached to a 25 mm square piece of clear PVC plastic using 5 mm thin iron wires, following the methodology by Yamazaki et al. (2020). In doing so, live prey were securely tethered but retained some capacity to display activity. The day before each experimental day, we prepared (a) 100 live prey of about 20 mm long, (b) 100 plasticine, 20 mm‐long, worm‐shaped prey, or (c) a half‐half mix of live prey and plasticine prey (50 live prey and 50 plasticine prey). Live prey were stored in a refrigerator set to 2°C to immobilize them while keeping them alive. Artificial prey were stored in a dark and cool environment to prevent their deterioration.

### 2.3 Experimental Procedure

We provided sentinel prey in similarly open habitats with sparse trees across the 10 selected study sites. Prey were deployed in one tree or shrub for 5 h between 07:00 h and 12:00 h (China Standard Time [UTC + 8 h]). The procedure was conducted for four continuous days (hereafter Day 1 to Day 4). On the first day, we deployed 100 live prey to attract birds (Yamazaki et al. 2020). From the second to the fourth day, 100 prey were deployed in three mixtures of live to plasticine prey. These mixtures consisted of either: (i) all live prey, (ii) all plasticine prey, or (iii) half live and half plasticine prey on each experimental day. The order of the mixtures for the final 3 days was randomized for each site over the final 3 days of this experiment. Prey were evenly distributed from the ground to the canopy of a shrub or tree, following a vertical line with 50‐cm intervals ranging from ground level to 2 m. Accordingly, 25 prey were secured at each interval. All prey were securely attached to branches and leaves using iron wires, with their orientation facing upward.

To accurately identify the predators involved in our experiment, we installed two camera traps (model: UVS5, China) to monitor the predators that interacted with our prey throughout the duration of the experiment. This method is applicable to a range of habitats and taxa (Moore et al. 2021). Cameras recorded continuously for 10 min with a 5‐s interval between recordings. These camera traps were positioned 1 m away from the releasing point and were vertically spaced 1 m apart from each other, ensuring the comprehensive coverage of all of the sentinel prey across the entire experiment. At the end of each experiment, we returned to the study site and collected all the prey and the cameras. In the field, we recorded the number of live prey that were fully consumed or partially eaten, as well as the number of plasticine prey that exhibited visible signs of being bitten.

**FIGURE 2:**
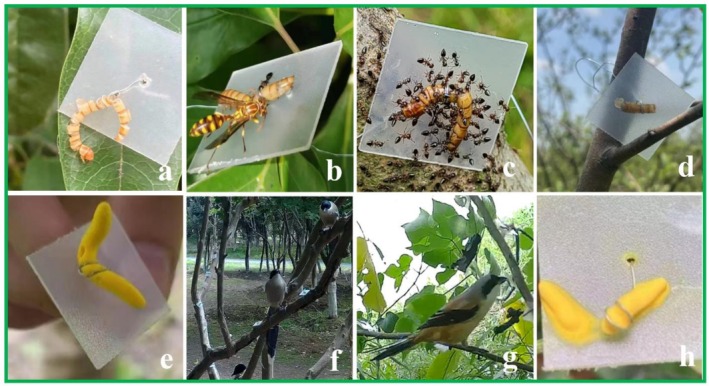
Images of predator identification. (a) Live prey consumed by wasps; (b) lesser paper wasp and (c) ants remaining on the prey; (d) live prey partially eaten by avian predators; (e) bite marks left by avian predators; (f) Azure‐winged magpie and (g) long‐tailed shrike were identified by the footage from camera traps; (h) plasticine prey melted by the rain and high temperature.

We attempted to identify the taxon of each predator through a combination of the following methods: bite marks left on the plasticine prey (Low et al. 2014), predators remaining on and around the prey when we collected them, and watching the footage captured by the camera traps. Wasp predation could be identified by a clean cut left on the live prey (Figure 2a). They were sometimes also recorded by our camera traps, but they were hard to identify to the species level. Wasps could also be identified if they remained on the prey when collecting the prey (Figure 2b). Ants could be identified because when they attacked the prey, they remained on the prey until the end of the experiment, and we were able to observe them (Figure 2c). When foragers are solitary, their presence may not be easily detected. To address this, we examined the aggregate scars on the prey. Avian predators could be identified by the remains of the live prey because the cuts left by birds were not as sharp as those left by wasps, and frequently there were stains on the PVC square (Figure 2d). The bite marks left on the plasticine prey from birds sometimes could be hard to discriminate from insect predators (Figure 2e), but birds were always captured by the camera traps (Figure 2f,g).

All the avian predators which attacked or consumed our prey belonged to Passeriformes, including Long‐tailed shrikes ( *Lanius schach* ), Oriental tits ( *Parus minor* ), Oriental Magpie‐Robins ( *Copsychus saularis* ), Azure‐winged magpies ( *Cyanopica cyanus* ), and Eurasian blackbirds ( *Turdus merula* ). Insect predators belonged to the order Hymenoptera and included Lesser paper wasps (*Parapolybia varia*) and multiple ant species (family Formicidae).

Predation intensity was estimated using the daily predation rate. At the end of each day, we recorded the number of live prey that were fully consumed or partially eaten, divided by the total number of live prey provided, as well as the number of plasticine prey that exhibited visible bite marks, divided by the total number of plasticine prey provided (Howe et al. 2009; Pena et al. 2021; Zvereva and Kozlov 2023). Predation rates were assessed separately for avian and insect predators. When no predation occurred, the predation rate was recorded as zero for both predator types. In cases where predation occurred from only one predator group, the predation rate was recorded as zero for the other group. The insect predation rate was excluded for the winter experiment since there was no insect predation due to the low temperatures characteristic of our study area.

Additionally, we aimed to assess the effects of inclement weather on artificial prey because artificial prey may be affected by extreme weather conditions such as high temperatures or rain (Muchula et al. 2019). To this aim, we selected a 5‐day exposure period to assess the risk of plasticine prey degrading due to rain or high temperatures. Of the 500 plasticine prey deployed, 135 (27%) of the plasticine prey melted due to rain and hot temperatures, which made the scars impossible to identify (Figure 2h), meaning that we excluded the results from this additional experiment.

### 2.4 Statistical Analysis

All the analyzes were carried out in R v 4.2.3 (R Core Team 2023), using the packages lme4 (Douglas et al. 2015), glmmTMB (Mollie et al. 2017), MuMin v 1.47.5 (Bartoń 2023), car v 3.1.2 (Fox and Weisberg 2019), DHARMa v 0.4.6 (Hartig 2022) and the figures were produced using ggplot2 (Wickham 2016).

To determine the impact that using artificial or live prey has on predation rate estimates, and how different predators responded to these two prey types, we built a generalized linear mixed model using predation rate, modeled as a proportion, as a binomial response variable in the Template Model Builder (glmmTMB) with the number of prey offered included as a weight. Experimental day number, urbanization rate, predator type, and the interaction between urbanization rate and predator type were explanatory variables, while site was included as a random factor. A zero‐inflation term was added to account for the high number of zeros observed in the data. Residual diagnostics showed no overdispersion with minor quantile deviations in the residuals compared with predicted values. We then ran model selection using the dredge function to find the best model, which included all the variables (see Table S1). The variance inflation factors (VIF) for all the predictors were lower than 3 except for predator type and the interaction between predator type and urbanization rate. Despite this issue, which is common when including interaction terms in the models, we retained these two variables in our model (Kock and Lynn 2012) (see Table S1). We also assessed spatial autocorrelation in the model residuals. We ran a Moran's I test using the spdep package, which yielded a score of 0.15 (*p* = 0.055), indicating non‐significant spatial autocorrelation (Bivand and Wong 2018).

## 3 Results

### 3.1 Live Versus Artificial Prey Performance

**FIGURE 3:**
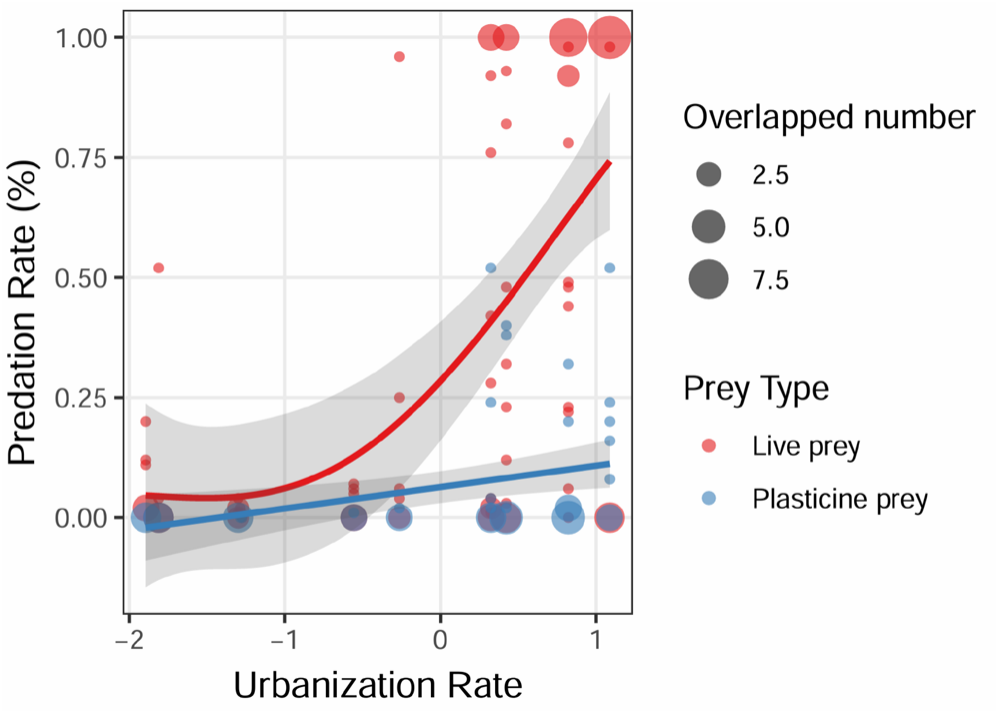
Estimation of predation rate using live prey and plasticine prey. Each dot represents the predation rate of the daily experiment using different types of prey (with 95% confidence intervals in gray). Prey types are distinguished by color. Point size reflects the number of overlapping observations.

We found a total of 2124 out of 6400 (33.19%) prey (93.08% live prey and 6.92% plasticine prey) that were bitten or consumed during the daily experiment. Predation rates of live prey were much higher than those of plasticine prey (z = −23.673, *p* < 0.001; Table 1). For all predator types combined, the predation rate of live prey increased with urbanization rates, reaching 100% at high urbanization sites. With regard to artificial prey, the predation rate reached up to 50% in highly urbanized sites (Table 1, Figure 3).

**TABLE 1:** Summary of the results examining the effects of prey type (live prey or plasticine prey), urbanization, and predator type (bird or insect), experiment duration, and the interactions between urbanization rate and predator type on predation rate.

Parameters	Estimate	SE	*t*	*p*
Intercept (site)	−5.602	1.036	−5.405	< 0.001***
Urbanization rate	16.656	2.610	6.381	< 0.001***
Predator type	1.745	0.382	4.565	< 0.001***
Prey type	−4.958	0.209	−23.673	< 0.001***
Experimental day number	0.900	0.038	2.356	0.019*
Urbanization rate × Predator type	−8.593	0.988	−8.700	< 0.001***

* *p* < 0.05.

*** *p* < 0.001.

### 3.2 Predator Responses to Prey Types and Urbanization

We found that 1879 (88.5% of the prey attacked) of the sentinel prey was attacked by birds and 245 (11.5%) were attacked by insect predators. For the live prey, 1733 (87.7% of the live prey attacked) were attacked by birds, and 244 (12.4%) were attacked by insect predators. For the plasticine prey, 146 (99.3% of the plasticine prey attacked) were attacked by avian predators and only 1 prey (0.7%) was attacked by an insect.

**FIGURE 4:**
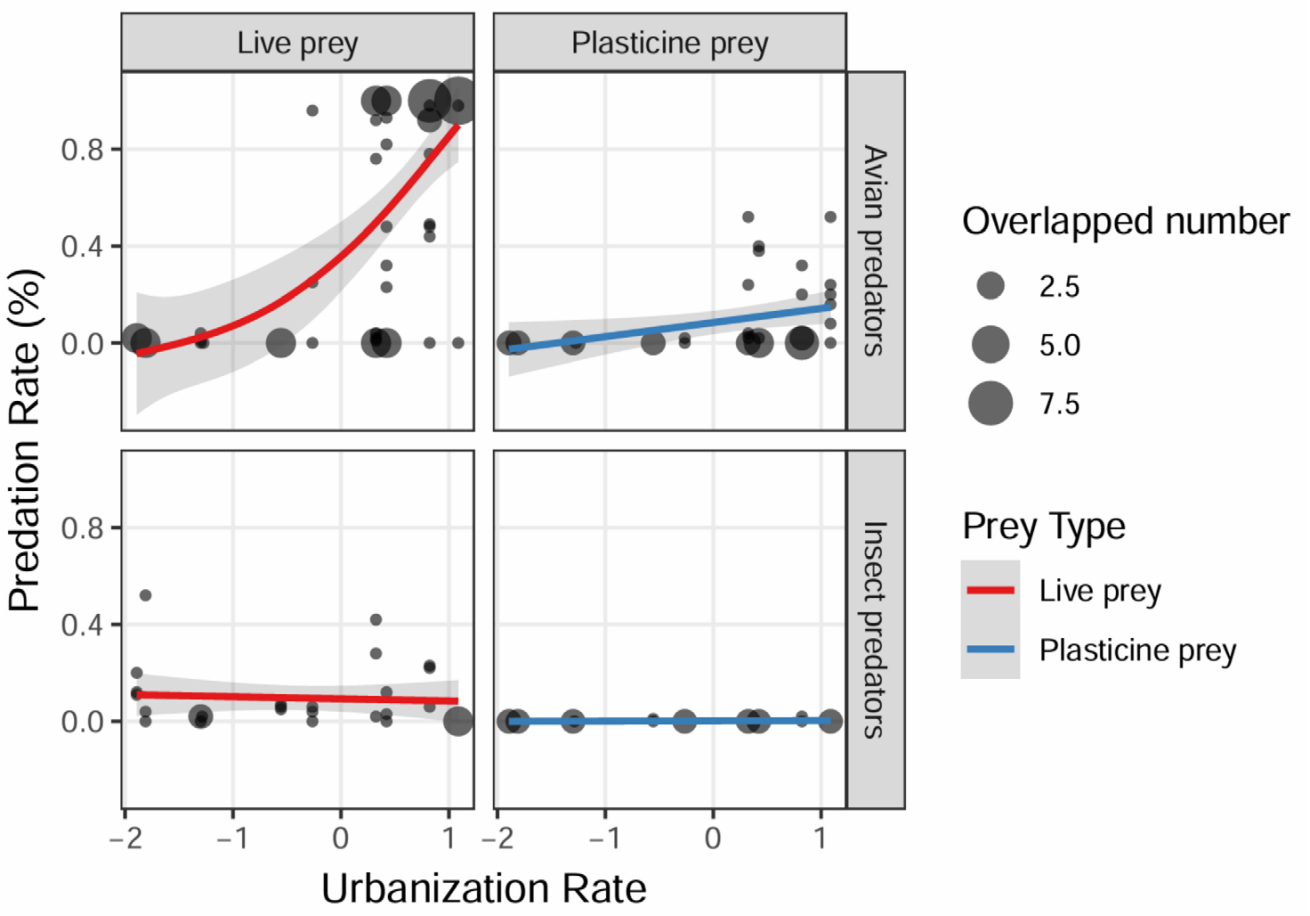
Estimation of predation rate estimated on live prey and plasticine prey for avian and insect predators. Each dot represents the predation rate of the daily experiment estimated on live or plasticine prey from bird and insect predators respectively, with (95% confidence intervals in gray). Point size reflects the number of overlapping observations.

**FIGURE 5:**
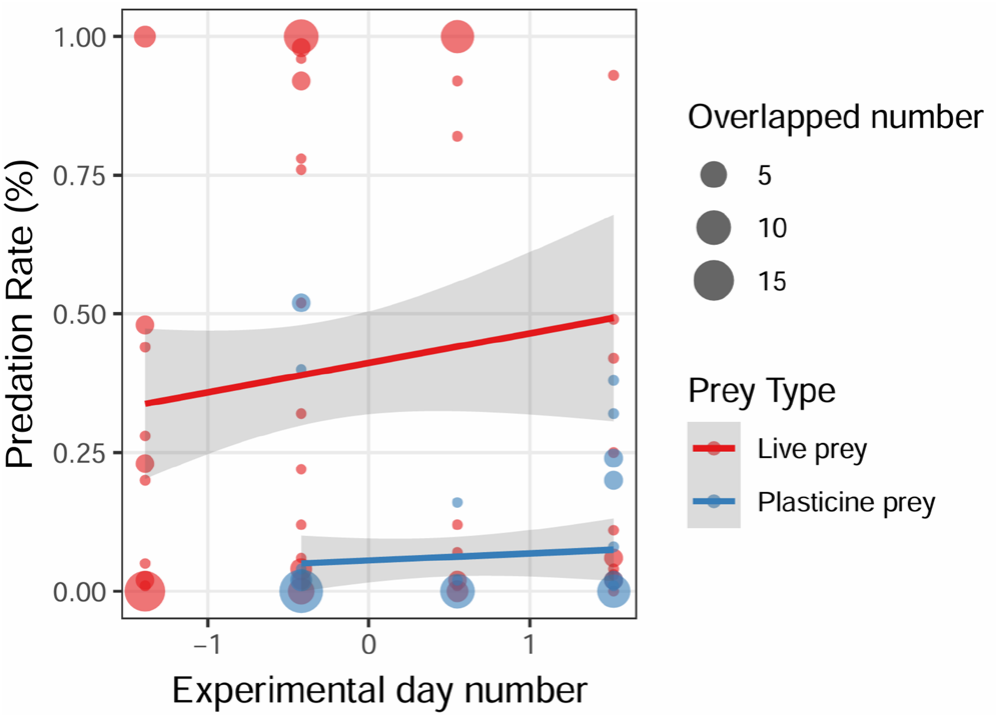
Comparison of predation rate estimated on live prey and plasticine prey with experimental day number. Each dot represents the predation rate of the daily experiment estimated on live or plasticine prey (95% confidence intervals in gray). Predation rate increased with experiment duration estimated on both types of prey. Point size reflects the number of overlapping observations.

When we examined predation along the urbanization gradient, we found that estimated predation rates differed for birds and insects (urbanization rate × predator type: z = −8.592, *p* < 0.001; Table 1). Avian predation rate and urbanization rate were correlated positively (estimated on both live prey and plasticine prey), with predation rate estimated on plasticine prey remaining at lower levels than the predation rate estimated on live prey, especially in highly urbanized sites (Figure 4). Conversely, insect predation intensity estimated on live prey correlated negatively with urbanization, while the predation rate estimated on plasticine prey was nearly absent along this urban gradient for both high and low urbanized sites compared with results from live prey (Figure 4). The predation rate estimated on both live prey and plasticine prey increased over the experimental day number (z = 2.356, *p* = 0.019; Table 1, Figure 5).

## 4 Discussion

In this study, we found that predation intensity on arthropods estimated using live prey was generally higher than that estimated on plasticine prey, especially in highly urbanized areas. While overall predation varied along the urbanization gradient, avian and insect predators showed contrasting patterns: avian predation increased with urbanization on both live and artificial prey, whereas insect predation declined with urbanization. Therefore, our results confirm that selecting the right prey type is crucial to accurately estimate predation intensity and that live and artificial prey tend to render different estimates of predation (Rodriguez‐Campbell et al. 2024; Zvereva et al. 2024).

### 4.1 Plasticine Prey and Live Prey Produce Different Predation Rate Estimates

In our study, the predation rate estimated on live prey was higher than that estimated on plasticine prey. One plausible explanation is that plasticine prey may be less appealing than live prey for predators that rely on olfactory cues, such as ants, due to differences in scent, making them less attractive (Manubay and Powell 2020). Plasticine prey may also fail to attract the attention of predators that rely on visual cues, such as birds (Martin 2012), as well as those that rely on a combination of multiple sensory cues, such as wasps (Mendes‐Silva et al. 2021). While the discernibility of live prey makes them more susceptible to detection, pursuit, and consumption by predators, consequently making predation estimates based on real prey higher than on artificial prey (Lövei and Ferrante 2017; Zou et al. 2017; Nimalrathna et al. 2023; Zvereva et al. 2024). Although we did not use dead prey in our experiment, it is plausible that predation rates on dead prey would lie between those observed for live and artificial prey (Rodriguez‐Campbell et al. 2024). Dead prey may keep some biological cues such as scent or appearance, but their lack of movement is likely to reduce the chances of being predated by visual predators like birds.

While plasticine prey may not fully replicate the predation pressure experienced by live prey, they remain a valuable tool for identifying predators (Low et al. 2014; Muchula et al. 2019). In fact, these two prey types had different benefits when determining predator identities. In our experiment, live prey had certain advantages, as wasps and ants often remained on the prey while it was collected, providing additional evidence of predation. Yet, live prey often left no traces or only stains on the PVC squares after being attacked by birds, making it difficult to identify predators without the footage from our camera traps (Moore et al. 2021). Although plasticine prey lack the inherent qualities that trigger the same level of predation intensity as live prey, the presence of visible marks enables relatively accurate predator identification (Low et al. 2014). This suggests that camera traps may need to be in place if using live prey in order to obtain an accurate representation of the predators present in the environment. Another notable limitation of using plasticine prey is that they may melt due to adverse weather conditions, including rain and high temperatures, resulting in the loss of identifiable scar data. This issue should be considered when adopting this method. There is also an ethical angle to this issue as researchers may wish not to use live prey for ethical reasons (e.g., not harming live animals) and thus artificial prey may be preferred, yet it would be necessary to acknowledge potential biases in estimates of predation derived from this approach.

### 4.2 Avian Predators Strongly Contributed to Predation Rate in Highly Urbanized Sites

Vertebrates can play a crucial role in regulating prey populations and controlling pests (Johnson et al. 2010; Sam et al. 2023; Sivault et al. 2024). Intuitively, in areas where there are fewer food resources, the predation rate on sentinel prey would be expected to be higher. While some studies have found that the predation rate increases with urbanization, mostly due to predator birds (Long and Frank 2020), other studies have failed to detect this trend (Eötvös et al. 2018; Pena et al. 2021). Our results suggest that previous studies might have failed to detect this pattern of increased predation rate with urbanization because many of these studies rely on plasticine prey to investigate this process. This raises concerns about the generality of trends based on artificial prey, as studies examining changes in predation pressure with latitude often rely solely on this prey type, potentially underestimating predation rates—especially for predators like birds, which can significantly contribute to overall predation rates (Ferrante et al. 2014; Roslin et al. 2017; Roquero et al. 2024). This pattern is consistent with findings from studies conducted in boreal regions, where live and artificial prey also produced divergent estimates of predation intensity (Zvereva and Kozlov 2023; Zvereva et al. 2024), although dead and live prey seem to show similar patterns (Rodriguez‐Campbell et al. 2024). Therefore, our and previous results suggest that when and where birds are expected to disproportionately contribute to overall predation rate or if the main goal is to perform comparisons across taxa, live or dead prey must be used preferentially (Rodriguez‐Campbell et al. 2024).

### 4.3 Predation Rate Estimated on Live Prey and Plasticine Prey Showed Similar Patterns for Avian but Not for Insect Predators Across an Urban Gradient

Urbanization involves profound environmental changes such as habitat fragmentation, loss of natural habitat, and biotic homogenization (McKinney 2006). Generalist predators may particularly benefit from these alterations as they can learn quickly to capitalize on new opportunities presented by these transformed landscapes and avoid inedible or toxic prey, like plasticine prey (Yamazaki et al. 2020; Morelli et al. 2018; Skelhorn and Rowe 2006; Marzluff et al. 2012). According to a previous study using both prey types, plasticine prey only attracted avian predators during the late breeding season once fledglings emerged from nests, possibly because they are less selective when foraging (Zvereva and Kozlov 2023). A similar pattern could be expected in highly urbanized areas, where food resources for predators are rather limited. In such environments, avian predators are more likely to exploit any available food source to survive, as increased competition can reduce their survival. This learning capacity of urban birds may allow them to quickly learn about the high profitability of live prey (Eisenhauer and Hines 2021), explaining why we recorded such a high predation rate by birds in highly urbanized sites. This could be supported by an increase in predation rates over the experiment days (see Figure 5), suggesting a potential learning effect by avian predators.

In contrast to birds, the intensity of predation by insect predators on live prey decreased along the urban gradient. One plausible explanation for this trend is related to the increasing disturbance hypothesis (Gray 1989), which suggests that the abundance of insect predators diminishes in urban environments. Insects may face challenges in adapting to the novel conditions associated with urbanization, such as exposure to pollutants, a reduction in green spaces, and altered microclimates (Campos et al. 2023). In our study, predation by insect predators on plasticine prey was nearly absent, suggesting that these models were not attracted to them. Since many insect predators rely on olfactory cues to locate prey, the absence of chemical signals in plasticine models may explain their low detection (Lövei and Ferrante 2017). Future studies could explore the use of scented or chemically treated models to better mimic live prey and improve their ecological realism.

It is important to note that our experiment focused on quantifying predation using worm‐shaped prey deployed during the day, which may bias observations toward visually oriented predators. Predators that are active at night or those that rely on other sensory cues or specialize in different prey morphologies were likely underrepresented, as significant predation pressure can occur during nocturnal hours (Berger and Gotthard 2008). Future studies could address this limitation by incorporating night monitoring or using a wider variety of prey types to capture a broader spectrum of predator interactions. Research has demonstrated that the richness of wasps and ants, two types of insect predators identified in our study, declines with increasing levels of urbanization (Buczkowski and Richmond 2012; Dürrbaum et al. 2022), which may be the main driver of the observed pattern. However, we did not assess the abundance of these predators or the various factors contributing to the effects of urbanization on them. Future studies should investigate the relationship between the abundance of potential predators and the quantification of predation intensity. Another limitation of this study is the potential overestimation of predation rates when using live prey. As the experiment progresses, birds may become more familiar with the prey's location, leading to increased exploitation over time. Further studies are needed to account for this learning effect and assess its impact on predation rate estimates.

## 5 Conclusion

Our analysis of predation pressure estimated on live and artificial prey underscores that avian and insect predators respond differently to live and artificial prey, rendering contrasting patterns of predation across an urban gradient. Previous research has indicated that using artificial prey can introduce biases when quantifying predation intensity, as they may not accurately represent the actual predation rate for different predators in the ecosystems (Zou et al. 2017; Greenop et al. 2019; Zvereva and Kozlov 2023; Zvereva et al. 2024). Nevertheless, one of the advantages of using plasticine prey is the possibility of identifying predator type from marks left on the prey (Low et al. 2014; Muchula et al. 2019). Our results suggest that artificial prey can be especially useful if discriminating predator identities is important for the study goals. However, it must be acknowledged that this approach may underestimate the true intensity of predation. Also, our results indicate that the use of camera traps might help to overcome this limitation for live prey, which highlights the potential advantages of combining field experiments with effective monitoring systems (Moore et al. 2021). While we have demonstrated that using different sentinel types can strongly influence predation rates, further research is needed to pinpoint the drivers of such divergence.

## Author Contributions

**Yu Zeng:** data curation (lead), formal analysis (supporting), methodology (lead), visualization (lead), writing – original draft (equal), writing – review and editing (supporting). **Haolin Yang:** investigation (supporting). **Yiheng Pan:** investigation (supporting). **Yuxuan Li:** investigation (supporting). **Dohee Kim:** investigation (supporting). **Haokun Wang:** investigation (supporting). **Jing Feng:** investigation (supporting). **Yuechen Huang:** investigation (supporting). **Yingjie Yin:** investigation (supporting). **Hanqing Zhao:** investigation (supporting). **Yuyang Wu:** investigation (supporting). **Craig R. A. Barnett:** conceptualization (equal), writing – review and editing (equal). **Catherine L. Parr:** conceptualization (equal), writing – review and editing (equal). **Samantha C. Patrick:** conceptualization (equal), writing – review and editing (equal). **Yi Zou:** conceptualization (equal), visualization (supporting), writing – review and editing (equal). **Emilio Pagani‐Núñez:** conceptualization (lead), formal analysis (lead), writing – original draft (equal), writing – review and editing (lead).

## Funding

This work was supported by Xi'an Jiaotong‐Liverpool University through the following grants: PGRS 1912013, RDF‐20‐01‐06, and SURF2021110.

## Conflicts of Interest

The authors declare no conflicts of interest.

## Supporting information


**Data S1:** ece372675‐sup‐0003‐Supinfo.docx.

## Data Availability

The data that support the findings of this study are publicly available in the Dryad Digital Repository at https://doi.org/10.5061/dryad.t76hdr8dz (Zeng et al. 2025).

## References

[ece372675-bib-0001] Alonso‐Crespo, I. M. , and J. A. Hernández‐Agüero . 2023. “Shedding Light on Trophic Interactions: A Field Experiment on the Effect of Human Population Between Latitudes on Herbivory and Predation Patterns.” Ecology and Evolution 13, no. 9: e10449. 10.1002/ece3.10449. 37664505 PMC10468994

[ece372675-bib-0002] Bartoń, K. 2023. “MuMIn: Multi‐Model Inference (R Package Version 1.47.5).” https://CRAN.R‐project.org/package=MuMIn.

[ece372675-bib-0003] Berger, D. , and K. Gotthard . 2008. “Time Stress, Predation Risk and Diurnal–Nocturnal Foraging Trade‐Offs in Larval Prey.” Behavioral Ecology and Sociobiology 62: 1655–1663. 10.1007/s00265-008-0594-4.

[ece372675-bib-0004] Birkhofer, K. , H. Bylund , P. Dalin , et al. 2017. “Methods to Identify the Prey of Invertebrate Predators in Terrestrial Field Studies.” Ecology and Evolution 7, no. 6: 1942–1953. 10.1002/ece3.2791. 28331601 PMC5355183

[ece372675-bib-0005] Bivand, R. , and D. Wong . 2018. “Comparing Implementations of Global and Local Indicators of Spatial Association.” Test 27, no. 3: 716–748. 10.1007/s11749-018-0599-x.

[ece372675-bib-0006] Buczkowski, G. , and D. Richmond . 2012. “The Effect of Urbanization on Ant Abundance and Diversity: A Temporal Examination of Factors Affecting Biodiversity.” PLoS One 7, no. 8: e41729. 10.1371/journal.pone.0041729. 22876291 PMC3410901

[ece372675-bib-0007] Campos, S. , S. Manes , G. Khattar , et al. 2023. “Global Meta‐Analysis of Urbanization Stressors on Insect Abundance, Richness, and Traits.” Science of the Total Environment 903: 165967. 10.1016/j.scitotenv.2023.165967. 37543317

[ece372675-bib-0008] Cupitra‐Rodríguez, J. , L. Cruz‐Bernate , and J. Montoya‐Lerma . 2023. “Attack Rates on Artificial Caterpillars in Urban Areas Are Higher Than in Suburban Areas in Colombia.” Journal of Tropical Ecology 39: e19. 10.1017/S026646742300007X.

[ece372675-bib-0009] Douglas, B. , M. Martin , B. Ben , and W. Steve . 2015. “Fitting Linear Mixed‐Effects Models Using lme4.” Journal of Statistical Software 67, no. 1: 1–48. 10.18637/jss.v067.i01.

[ece372675-bib-0010] Dürrbaum, E. , F. Fornoff , C. Scherber , E. Vesterinen , and B. Eitzinger . 2022. “Metabarcoding of Trap Nests Reveals Differential Impact of Urbanization on Cavity‐Nesting Bee and Wasp Communities.” Molecular Ecology 32: 1369–1383. 10.1111/mec.16818. 36479967

[ece372675-bib-0011] Eisenhauer, N. , and J. Hines . 2021. “Invertebrate Biodiversity and Conservation.” Current Biology 31, no. 24: R1214–R1218. 10.1016/j.cub.2021.06.058. 34637734

[ece372675-bib-0012] Eötvös, C. B. , T. Magura , and G. L. Lövei . 2018. “A Meta‐Analysis Indicates Reduced Predation Pressure With Increasing Urbanization.” Landscape and Urban Planning 180: 54–59. 10.1016/j.landurbplan.2018.08.010.

[ece372675-bib-0013] Fenoglio, M. S. , M. R. Rossetti , and M. Videla . 2020. “Negative Effects of Urbanization on Terrestrial Arthropod Communities: A Meta‐Analysis.” Global Ecology and Biogeography 29, no. 8: 1412–1429. 10.1111/geb.13107.

[ece372675-bib-0014] Ferrante, M. , A. Lo Cacciato , and G. L. Lövei . 2014. “Quantifying Predation Pressure Along an Urbanisation Gradient in Denmark Using Artificial Caterpillars.” European Journal of Entomology 111, no. 5: 649–654. 10.14411/eje.2014.082.

[ece372675-bib-0015] Fox, J. , and S. Weisberg . 2019. An R Companion to Applied Regression. 3rd ed. Sage Publications. https://socialsciences.mcmaster.ca/jfox/Books/Companion/.

[ece372675-bib-0016] Gering, J. C. , and R. B. Blair . 1999. “Predation on Artificial Bird Nests Along an Urban Gradient: Predatory Risk or Relaxation in Urban Environments?” Ecography 22, no. 5: 532–541. 10.1111/j.1600-0587.1999.tb01283.x.

[ece372675-bib-0017] Gilbert, S. , N. Carter , and R. Naidoo . 2021. “Predation Services: Quantifying Societal Effects of Predators and Their Prey.” Frontiers in Ecology and the Environment 19, no. 5: 292–299. 10.1002/fee.2336.

[ece372675-bib-0018] Gray, J. S. 1989. “Effects of Environmental Stress on Species Rich Assemblages.” Biological Journal of the Linnean Society 37, no. 1–2: 19–32. 10.1111/j.1095-8312.1989.tb02003.x.

[ece372675-bib-0019] Greenop, A. , A. Cecelja , B. A. Woodcock , A. Wilby , S. M. Cook , and R. F. Pywell . 2019. “Two Common Invertebrate Predators Show Varying Predation Responses to Different Types of Sentinel Prey.” Journal of Applied Entomology 143, no. 4: 380–386. 10.1111/jen.12612.

[ece372675-bib-0020] Guo, F. , T. C. Bonebrake , and C. Dingle . 2016. “Low Frequency Dove Coos Vary Across Noise Gradients in an Urbanized Environment.” Behavioural Processes 129: 86–93. 10.1016/j.beproc.2016.06.013. 27268468

[ece372675-bib-0021] Hartig, F. 2022. “DHARMa: Residual Diagnostics for Hierarchical (Multi‐Level/Mixed) Regression Models (R Package Version 0.4.6).” https://CRAN.R‐project.org/package=DHARMa.

[ece372675-bib-0022] He, R. , E. Pagani‐Núñez , E. Goodale , and C. R. A. Barnett . 2022. “Avian Predators Taste Reject Mimetic Prey in Relation to Their Signal Reliability.” Scientific Reports 12, no. 1: 2334. 10.1038/s41598-022-05600-5. 35149707 PMC8837650

[ece372675-bib-0023] Houska, T. M. , O. Mottl , L. R. Jorge , B. Koane , V. Novotny , and K. Sam . 2023. “Trophic Cascades in Tropical Rainforests: Effects of Vertebrate Predator Exclusion on Arthropods and Plants in Papua New Guinea.” Biotropica 55, no. 1: 70–80. 10.1111/btp.13160.

[ece372675-bib-0024] Howe, A. , G. L. Lövei , and G. Nachman . 2009. “Dummy Caterpillars as a Simple Method to Assess Predation Rates on Invertebrates in a Tropical Agroecosystem.” Entomologia Experimentalis et Applicata 131, no. 3: 325–329. 10.1111/j.1570-7458.2009.00860.x.

[ece372675-bib-0025] Johnson, M. D. , J. L. Kellermann , and A. M. Stercho . 2010. “Pest Reduction Services by Birds in Shade and Sun Coffee in Jamaica.” Animal Conservation 13, no. 2: 140–147. 10.1111/j.1469-1795.2009.00310.x.

[ece372675-bib-0102] Kock, N. , and G. Lynn . 2012. “Lateral Collinearity and Misleading Results in Variance‐Based SEM: An Illustration and Recommendations.” Journal of the Association of Information Systems 13: 546–580.

[ece372675-bib-0026] Korányi, D. , M. H. Egerer , A. Rusch , B. Szabó , and P. Batáry . 2022. “Urbanization Hampers Biological Control of Insect Pests: A Global Meta‐Analysis.” Science of the Total Environment 834: 155396. 10.1016/j.scitotenv.2022.155396. 35460770

[ece372675-bib-0027] Kozlov, M. V. , V. Lanta , V. Zverev , K. Rainio , M. A. Kunavin , and E. L. Zvereva . 2017. “Decreased Losses of Woody Plant Foliage to Insects in Large Urban Areas Are Explained by Bird Predation.” Global Change Biology 23, no. 10: 4354–4364. 10.1111/gcb.13692. 28317226

[ece372675-bib-0028] Lidasan, A. K. , J. O. Roquero , N. K. B. Balasa , A. R. Agduma , R. J. A. Ele , and K. C. Tanalgo . 2023. “Predator Types, Urbanization, and Tree Cover Drive Top‐Down Control of Herbivorous and Carnivorous Preys in an Urban Agroecosystem.” Écoscience 30, no. 2: 158–168. 10.1080/11956860.2023.2244301.

[ece372675-bib-0029] Long, L. C. , and S. D. Frank . 2020. “Risk of Bird Predation and Defoliating Insect Abundance Are Greater in Urban Forest Fragments Than Street Trees.” Urban Ecosystems 23: 1125–1133. 10.1007/s11252-020-00939-x.

[ece372675-bib-0030] Lövei, G. L. , and M. Ferrante . 2017. “A Review of the Sentinel Prey Method as a Way of Quantifying Invertebrate Predation Under Field Conditions: Measuring Predation Pressure by Sentinel Prey.” Insect Science 24, no. 4: 528–542. 10.1111/1744-7917.12405. 27686246

[ece372675-bib-0031] Low, P. A. , K. Sam , C. McArthur , M. R. C. Posa , and D. F. Hochuli . 2014. “Determining Predator Identity From Attack Marks Left in Model Caterpillars: Guidelines for Best Practice.” Entomologia Experimentalis et Applicata 152, no. 2: 120–126. 10.1111/eea.12207.

[ece372675-bib-0032] Manubay, J. , and S. Powell . 2020. “Detection of Prey Odours Underpins Dietary Specialization in a Neotropical Top Predator: How Army Ants Find Their Ant Prey.” Journal of Animal Ecology 89, no. 9: 1980–1991. 10.1111/1365-2656.13188. 32097493

[ece372675-bib-0033] Martin, G. R. 2012. “Through Birds' Eyes: Insights Into Avian Sensory Ecology.” Journal of Ornithology 153, no. S1: 23–48. 10.1007/s10336-011-0771-5.

[ece372675-bib-0103] Marzluff, J. M. , R. Bowman , and R. Donnelly . 2012. “Avian Ecology and Conservation in an Urbanizing World.” Auk 119: 889–892.

[ece372675-bib-0034] McKinney, M. L. 2006. “Urbanization as a Major Cause of Biotic Homogenization.” Biological Conservation 127, no. 3: 247–260. 10.1016/j.biocon.2005.09.005.

[ece372675-bib-0035] Mendes‐Silva, I. , D. Queiroga , E. Calixto , H. M. Torezan‐Silingardi , and K. Del‐Claro . 2021. “Multiple Cues Guarantee Successful Predation by a Neotropical Wasp.” Behaviour 158, no. 1: 25–43. 10.1163/1568539X-bja10144.

[ece372675-bib-0036] Mollie, E. B. , K. Kasper , J. v. B. Koen , et al. 2017. “glmmTMB Balances Speed and Flexibility Among Packages for Zero‐Inflated Generalized Linear Mixed Modeling.” R Journal 9, no. 2: 378–400. 10.32614/RJ-2017-066.

[ece372675-bib-0037] Moore, J. F. , K. Soanes , D. Balbuena , et al. 2021. “The Potential and Practice of Arboreal Camera Trapping.” Methods in Ecology and Evolution 12, no. 10: 1768–1779. 10.1111/2041-210X.13666.

[ece372675-bib-0038] Morelli, F. , P. Mikula , Y. Benedetti , R. Bussière , L. Jerzak , and P. Tryjanowski . 2018. “Escape Behaviour of Birds in Urban Parks and Cemeteries Across Europe: Evidence of Behavioural Adaptation to Human Activity.” Science of the Total Environment 631‐632: 803–810. 10.1016/j.scitotenv.2018.03.118. 29727990

[ece372675-bib-0039] Muchula, K. , G. Xie , and G. M. Gurr . 2019. “Ambient Temperature Affects the Utility of Plasticine Caterpillar Models as a Tool to Measure Activity of Predators Across Latitudinal and Elevational Gradients.” Biological Control 129: 12–17. 10.1016/j.biocontrol.2018.11.006.

[ece372675-bib-0040] Nimalrathna, T. S. , I. D. Solina , A. M. Mon , et al. 2023. “Estimating Predation Pressure in Ecological Studies: Controlling Bias Imposed by Using Sentinel Plasticine Prey.” Entomologia Experimentalis et Applicata 171, no. 1: 56–67. 10.1111/eea.13249.

[ece372675-bib-0041] Pena, J. C. , F. Aoki‐Gonçalves , W. Dáttilo , M. C. Ribeiro , and I. MacGregor‐Fors . 2021. “Caterpillars' Natural Enemies and Attack Probability in an Urbanization Intensity Gradient Across a Neotropical Streetscape.” Ecological Indicators 128: 107851. 10.1016/j.ecolind.2021.107851.

[ece372675-bib-0042] R Core Team . 2023. R: A Language and Environment for Statistical Computing. R Foundation for Statistical Computing. https://www.R‐project.org/.

[ece372675-bib-0043] Rodriguez‐Campbell, A. , O. Rahn , M. C. Chiuffo , and A. L. Hargreaves . 2024. “Clay Larvae Do Not Accurately Measure Biogeographic Patterns in Predation.” Journal of Biogeography 51, no. 6: 1004–1013. 10.1111/jbi.14800.

[ece372675-bib-0044] Roquero, J. , A. Lidasan , K. Balasa‐Navel , et al. 2024. “Predation Risk of Caterpillar Prey Is Shaped by Arthropods and Urbanisation in an Urban‐Agricultural Landscape: A Common Garden Experiment.” Urban Ecosystems 27, no. 6: 2267–2276. 10.1007/s11252-024-01587-1.

[ece372675-bib-0045] Roslin, T. , B. Hardwick , V. Novotny , et al. 2017. “Higher Predation Risk for Insect Prey at Low Latitudes and Elevations.” Science 356, no. 6339: 742–744. 10.1126/science.aaj1631. 28522532

[ece372675-bib-0046] Sam, K. , L. R. Jorge , B. Koane , P. K. Amick , and E. Sivault . 2023. “Vertebrates, but Not Ants, Protect Rainforest From Herbivorous Insects Across Elevations in Papua New Guinea.” Journal of Biogeography 50, no. 10: 1803–1816. 10.1111/jbi.14686.

[ece372675-bib-0047] Shochat, E. , P. S. Warren , S. H. Faeth , N. E. McIntyre , and D. Hope . 2006. “From Patterns to Emerging Processes in Mechanistic Urban Ecology.” Trends in Ecology & Evolution 21, no. 4: 186–191. 10.1016/j.tree.2005.11.019. 16701084

[ece372675-bib-0048] Sivault, E. , J. Kollross , L. Jorge , et al. 2024. “Insectivorous Birds and Bats Outperform Ants in the Top‐Down Regulation of Arthropods Across Strata of a Japanese Temperate Forest.” Journal of Animal Ecology 93, no. 11: 1622–1638. 10.1111/1365-2656.14146. 39045801

[ece372675-bib-0049] Skelhorn, J. , and C. Rowe . 2006. “Avian Predators Taste–Reject Aposematic Prey on the Basis of Their Chemical Defence.” Biology Letters 2, no. 3: 348–350. 10.1098/rsbl.2006.0483. 17148400 PMC1686200

[ece372675-bib-0050] Taylor, R. J. 2013. Predation. Springer.

[ece372675-bib-0101] Wang, L. , J. Shen , and C. K. L. Chung . 2015. “City Profile: Suzhou – A Chinese City Under Transformation.” Cities 44: 60–72.

[ece372675-bib-0051] Wickham, H. 2016. ggplot2: Elegant Graphics for Data Analysis. Springer‐Verlag.

[ece372675-bib-0052] Yamazaki, Y. , E. Pagani‐Núñez , T. Sota , and C. R. A. Barnett . 2020. “The Truth Is in the Detail: Predators Attack Aposematic Prey With Less Aggression Than Other Prey Types.” Biological Journal of the Linnean Society 131, no. 2: 332–343. 10.1093/biolinnean/blaa119.

[ece372675-bib-0104] Zeng, Y. , H. Yang , Y. Pan , et al. 2025. “Testing the Sentinel Method: Live and Artificial Prey Display Contrasting Patterns of Predation Across an Urban Gradient.” Dryad Digital Repository. 10.5061/dryad.t76hdr8dz. PMC12711592 41426649

[ece372675-bib-0053] Zou, Y. , J. de Kraker , F. J. J. A. Bianchi , M. D. van Telgen , H. Xiao , and W. van der Werf . 2017. “Video Monitoring of Brown Planthopper Predation in Rice Shows Flaws of Sentinel Methods.” Scientific Reports 7, no. 1: 42210. 10.1038/srep42210. 28211500 PMC5314450

[ece372675-bib-0054] Zvereva, E. L. , B. Adroit , T. Andersson , et al. 2024. “Predation on Live and Artificial Insect Prey Shows Different Global Latitudinal Patterns.” Global Ecology and Biogeography 33, no. 11: e13899. 10.1111/geb.13899.

[ece372675-bib-0055] Zvereva, E. L. , and M. V. Kozlov . 2021. “Latitudinal Gradient in the Intensity of Biotic Interactions in Terrestrial Ecosystems: Sources of Variation and Differences From the Diversity Gradient Revealed by Meta‐Analysis.” Ecology Letters 24, no. 11: 2506–2520. 10.1111/ele.13851. 34322961

[ece372675-bib-0056] Zvereva, E. L. , and M. V. Kozlov . 2023. “Predation Risk Estimated on Live and Artificial Insect Prey Follows Different Patterns.” Ecology 104, no. 3: e3943. 10.1002/ecy.3943. 36477626

