# Supplementary material for: Testing the Sentinel Method: Live and Artificial Prey Display Contrasting Patterns of Predation Across an Urban Gradient
Source: Ecol Evol. 2025 Dec 17;15(12):e72675. doi: 10.1002/ece3.72675 (PMC12711592; doi:10.1002/ece3.72675)
Supplement: Supplementary file 1 — Data S1: ece372675‐sup‐0003‐Supinfo.docx. [file ECE3-15-e72675-s001.docx]

**Supplement**

Title: Testing the sentinel method: live and artificial prey display contrasting patterns of predation across an urban gradient

Yu Zeng^1^, Haolin Yang^1^, Yiheng Pan^1^, Yuxuan Li^2^, Dohee Kim^3^, Haokun Wang^1^, Jing Feng^4^, Yuechen Huang^1^, Yingjie Yin^3^, Hanqing Zhao^1^, Yuyang Wu^1^, Craig R. A. Barnett^5^, Catherine L. Parr^6,7,8^, Samantha Patrick^6^, Yi Zou^1^, Emilio Pagani-Núñez^1,9,10^*

^1^ Department of Health and Environmental Sciences, School of Science, Xi’an Jiaotong-Liverpool University, Suzhou, China

^2^ School of Advanced Technology, Xi’an Jiaotong-Liverpool University, Suzhou, China

^3^ Department of Biosciences and Bioinformatics, Xi’an Jiaotong-Liverpool University, Suzhou, China

^4^ School of Advanced Technology, Xi’an Jiaotong-Liverpool University, Suzhou, China

^5^ Department of Zoology, Graduate School of Science, Kyoto University, Kyoto, Japan

^6^ Department of Earth, Ocean and Ecological Sciences, School of Environmental Science, University of Liverpool, Liverpool, UK

^7^ Department of Zoology & Entomology, University of Pretoria, Pretoria, South Africa

^8^ School of Animal, Plant and Environmental Sciences, University of the Witwatersrand, Wits, South Africa

^9^ Centre for Conservation and Restoration Science, Edinburgh Napier University, Edinburgh, UK

^10^ School of Applied Sciences, Edinburgh Napier University, Edinburgh, UK

* corresponding author: [e.pagani-nunez@napier.ac.uk](mailto:e.pagani-nunez@napier.ac.uk)

Research Article for *Ecology and Evolution*

Context

**Site information1**

Table S1: Site information and urbanization quantification.2

**Experiment design3**

**Models and results5**

Table S2. Variance Inflation Factors (VIF), Tolerance, and Correlation Level for Predictor Variables in the Model5

Figure S1 Distribution of predation rate. Multiple zero values showed in the figure

6

Figure S2 Diagnostic plots for model residuals.7

Table S3: Models selection8

**Site information:**

The experiment was conducted in ten sites following an urbanization gradient from natural reserves, agricultural farmlands to urban parks from December, 2020 to August, 2021.

Each site was extracted from the original LULC map within a standard circle with a radius of one kilometer and centered at the central point. The urbanization rate of each site was quantified using built area dividing the whole area, which the water area was excluded since there are large potation of water area in some sites which could be a bias.

Table S1: Site information and urbanization quantification.

| **Category** | **Site**  **Land use** | **Tongli wetland Park** | **Yundong Park** | **Yangjia**  **Village** | **Lingyan**  **Mountain** | **Bailu**  **Park** | **Jiangtian**  **Village** | **Dongshahu**  **Park** | **Dushuhu**  **Park** | **Yangchenghu**  **Orchad** | **Chenghu**  **Farm** |
| --- | --- | --- | --- | --- | --- | --- | --- | --- | --- | --- | --- |
| **1** | **Trees** | **6324** | **3744** | **6393** | **25466** | **7777** | **1158** | **7656** | **1891** | **5812** | **2494** |
| **2** | **Scrub/shrub** | **79** | **23** | **52** |  | **56** | **17** | **74** |  | **76** | **8** |
| **3** | **Grass** | **398** | **1452** | **65** | **212** | **1315** | **255** | **3123** | **724** | **1511** | **563** |
| **4** | **Crops** | **17272** | **5166** | **21977** | **2867** | **1903** | **15062** | **4438** | **4141** | **6908** | **14921** |
| **5** | **Built-up** | **2562** | **14155** | **2621** | **5883** | **8544** | **8211** | **12059** | **12584** | **5959** | **3913** |
| **6** | **Bare ground** | **3402** | **5324** | **4795** | **2228** | **1737** | **1467** | **1721** | **5180** | **1705** | **1878** |
| **7** | **Ice/Snow** |  |  |  |  |  |  |  |  |  |  |
| **8** | **Water** | **6468** | **6790** | **671** |  | **15325** | **10469** | **7527** | **12108** | **14609** | **12831** |
| **9** | **Flooded vegetation** | **153** | **2** | **84** |  | **1** | **19** | **43** |  | **78** | **50** |
| **total** |  | **36658** | **36656** | **36658** | **36656** | **36658** | **36658** | **36641** | **36628** | **36658** | **36658** |
| **urbanization rate** | | **0.070** | **0.386** | **0.071** | **0.160** | **0.233** | **0.224** | **0.329** | **0.344** | **0.162** | **0.107** |
| **Urbanization rate (exclude water)** | | **0.085** | **0.474** | **0.0728** | **0.160** | **0.401** | **0.314** | **0.414** | **0.513** | **0.270** | **0.164** |

**Experiment design:**


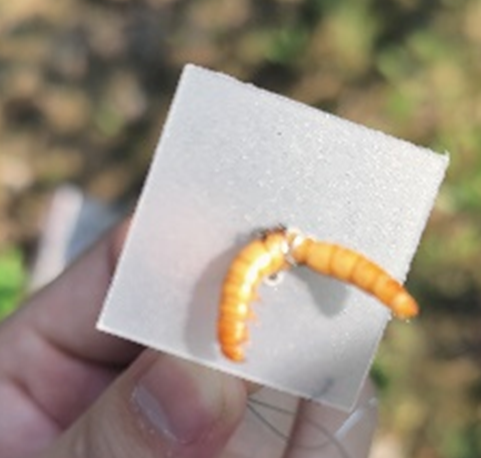

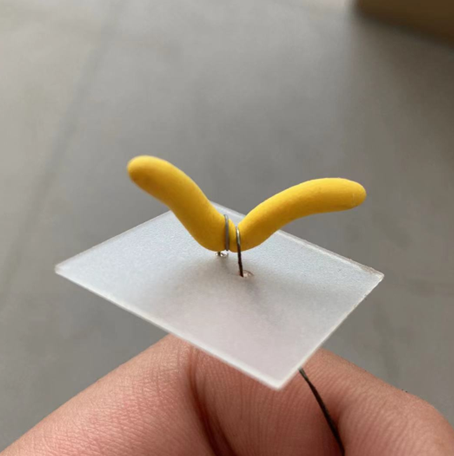
Site 1: We prepared a) 100 live prey of about 20mm long, or b) 100 plasticine, 20mm-long, worm-shaped models, or c) a half-half mix of live prey and plasticine models (50 live prey and 50 plasticine models).

Prey was evenly distributed from the ground to the canopy of a shrub or tree, following a vertical line with 50-centimeter intervals ranging from zero meters to two meters. Accordingly, twenty-five prey were secured to each interval. All prey were securely attached to sticks or leaves using iron wires, with their orientation facing upward.

Two camera traps were positioned 1 meter away from the releasing point and were vertically spaced 1 meter apart from each other, ensuring the comprehensive coverage of the experimental area


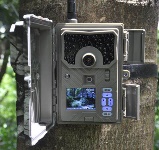

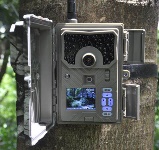

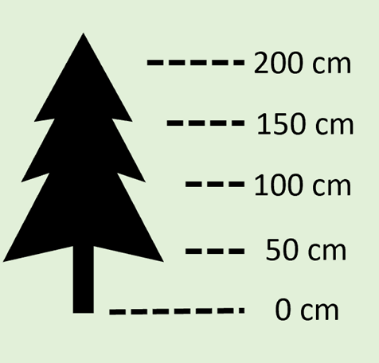

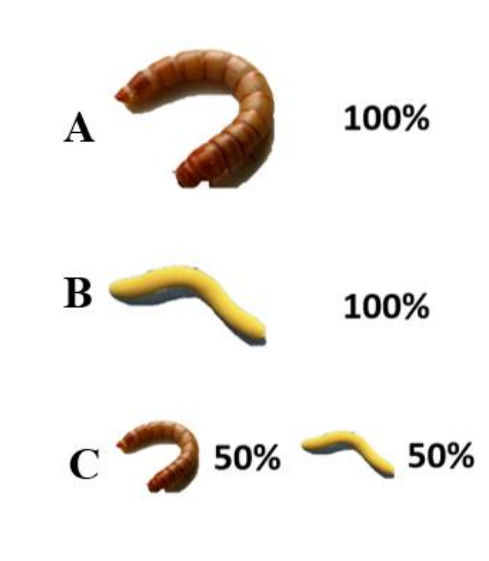


Prey was provided at 7 a.m. and left in the environment for a period of 5h until 12 p.m. The procedure was conducted in four continuous days, noted as Day 1 to 4.

Site 2:


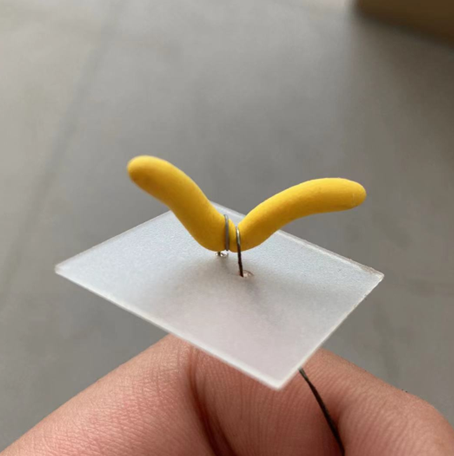
In the other location, we conducted the long-term experiment by which fifty plasticine models were exposed in the study site for four continuous days throughout the entire procedure. Prey was provided at 7 am on the first day and collected at 12 a.m on the fourth day.

x 50

**Data recording:**

Predation intensity was estimated using predation rates which were quantified daily for the short-term experiment. At the end of each day, we used the recorded the number of live prey that were fully consumed and partially eaten dividing the total live prey provided, as well as the number of plasticine models that exhibited visible signs of being bitten dividing the total plasticine model provided.

Predation rates were assessed separately for avian and arthropod predators based on the predators identified combining bite marks and video footages.

For the long-term experiment, predation intensity of each site was estimated using predation rates which were quantified using the number of the prey with bite marks left on the plasticine models diving the total number of the plasticine models provided on the first day.

Data sheet:

| Site | UrbanizationRate | DayN | PredationRate | PreyType | PredatorType | Number (predated) | Total (number released) |
| --- | --- | --- | --- | --- | --- | --- | --- |
|  |  | 1-4 | Number/Total | Live/plasticine | Insect/avian |  |  |

Since the response variable is proportion (predation rate) with many zeros (Figure S1), we built a zero-inflated binomial model. The explanatory variables include urbanization rate, predator type and interaction between them, plus prey type (Live or Plasticine prey), day number and site as random factor. Then we tested if the models fits well using DHARMa package, and no over dispersed issue was detected (Figure S2).

model1 <- glmmTMB(

PredationRate ~ UrbanizationRate * PredatorType + PreyType + DayN + (1 | Site),

ziformula = ~1,

weights = Total,

family = binomial(link = "logit"),

na.action = na.fail,

data = mydata

)


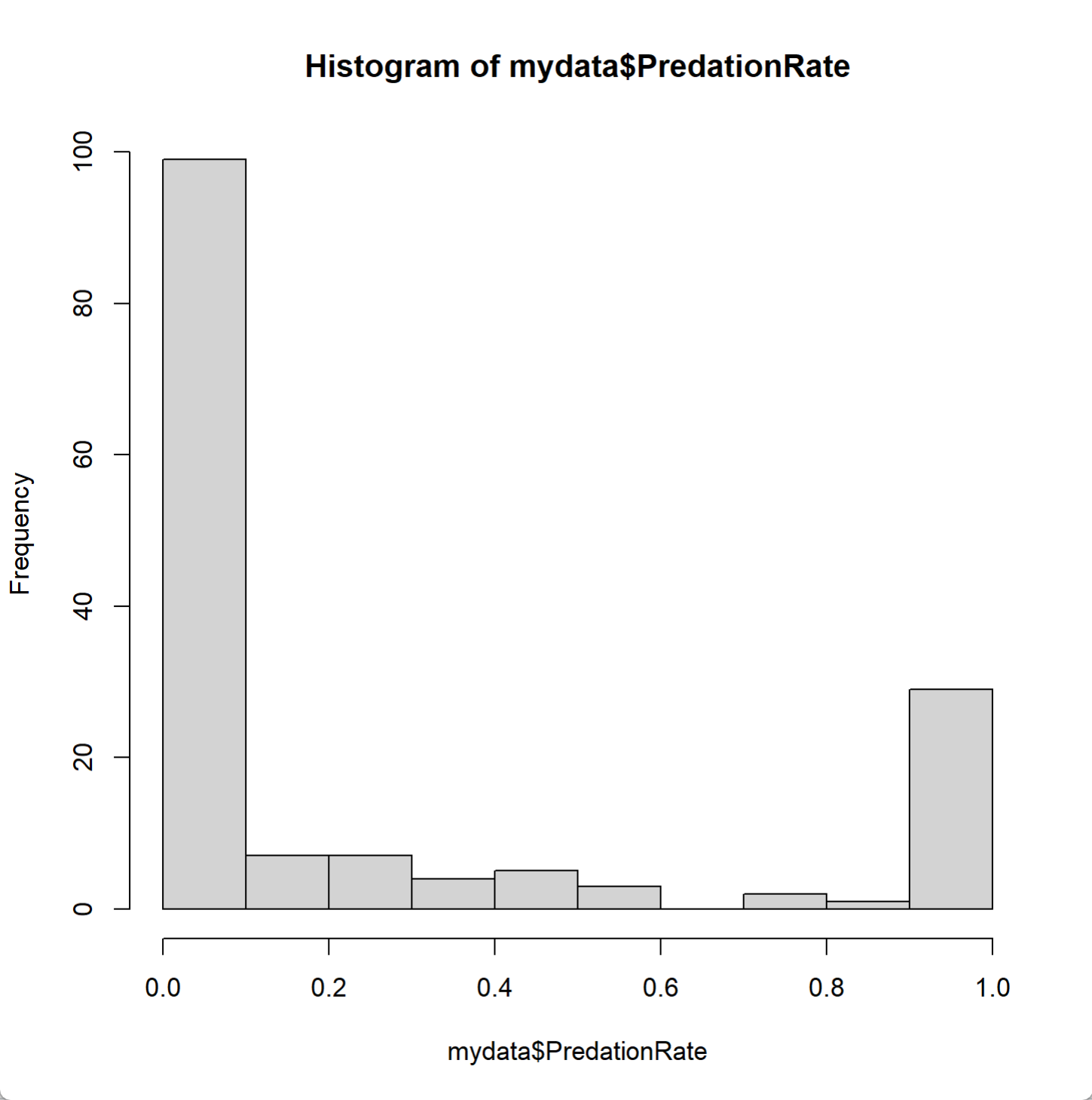


Figure S1 Distribution of predation rate.

Multiple zero values showed in the figure


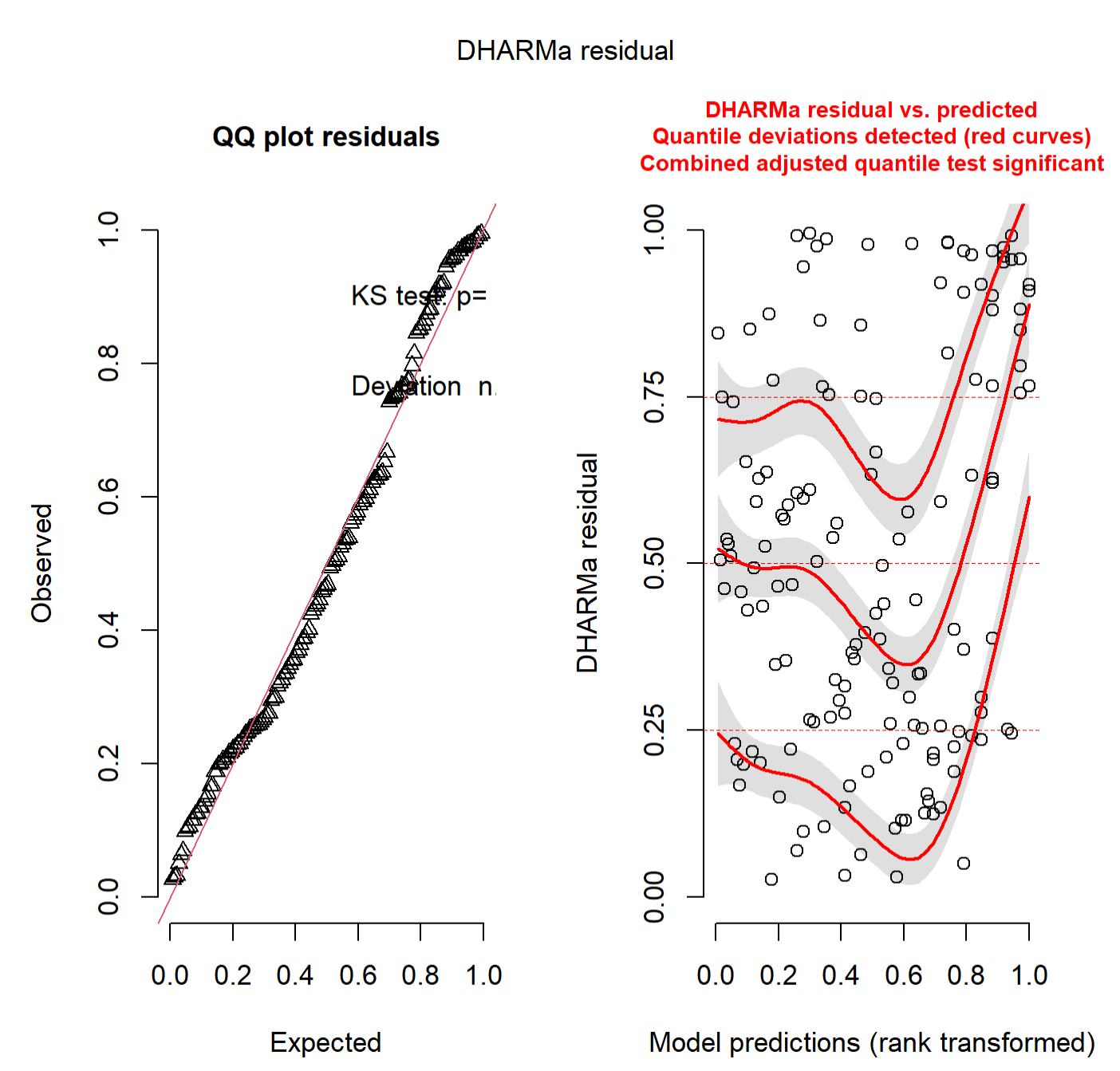


Figure S2 Diagnostic plots for model residuals.

The left panel shows a quantile–quantile (QQ) plot of the residuals with no significant Kolmogorov–Smirnov test (p = 0.333), indicating no deviation from uniformity. The right panel plots DHARMa residuals shows potential overdispersion in predicted values.

We want to choose the best model, so we conducted model selection (Dredge function in MuMIn package):

Table S2: Models selection, ranked by AICc value.

| Model | Cond. Intercept | ZI Intercept | Cond. DyN | Cond. PrdT | Cond. PryT | Cond. UrR | Cond. PrdT:UrR | df | logLik | AICc | Delta AICc | Model Weight |
| --- | --- | --- | --- | --- | --- | --- | --- | --- | --- | --- | --- | --- |
| 32 | -5.602 | -1.127 | + | + | + | + | + | 8 | -694.858 | 1406.8 | 0 | 0.841 |
| 31 | -5.396 | -1.115 |  | + | + | + | + | 7 | -697.644 | 1410.1 | 3.34 | 0.159 |
| 16 | -3.631 | -1.05 | + | + | + | + |  | 7 | -738.24 | 1491.3 | 84.53 | 0 |
| 15 | -3.471 | -1.04 |  | + | + | + |  | 6 | -740.299 | 1493.2 | 86.44 | 0 |
| 8 | 0.8956 | -1.051 | + | + | + |  |  | 6 | -745.257 | 1503.1 | 96.36 | 0 |
| 7 | 1.071 | -1.042 |  | + | + |  |  | 5 | -747.343 | 1505.1 | 98.36 | 0 |
| 13 | -4.824 | -1.058 |  |  |  | + |  | 5 | -828.541 | 1667.5 | 260.75 | 0 |
| 14 | -4.899 | -1.062 | + |  |  | + |  | 6 | -828.139 | 1668.9 | 262.12 | 0 |
| 5 | 0.6499 | -1.06 |  |  |  |  |  | 4 | -836.746 | 1681.8 | 275.02 | 0 |
| 6 | 0.5726 | -1.064 | + |  |  |  |  | 5 | -836.344 | 1683.1 | 276.36 | 0 |
| 28 | -3.918 | -0.4369 | + | + |  |  | + | 7 | -1303.2 | 2621.2 | 1214.44 | 0 |
| 12 | -2.137 | -0.4153 | + | + |  |  |  | 6 | -1334.73 | 2682.1 | 1275.3 | 0 |
| 4 | 0.7713 | -0.416 | + | + |  |  |  | 5 | -1341.42 | 2693.3 | 1286.5 | 0 |
| 27 | -4.591 | -0.4309 |  | + |  |  | + | 6 | -1366.43 | 2745.4 | 1338.7 | 0 |
| 10 | -3.213 | -0.4352 |  |  |  |  |  | 5 | -1386.68 | 2783.8 | 1377.02 | 0 |
| 2 | 0.4718 | -0.438 | + |  |  |  |  | 4 | -1394.82 | 2797.9 | 1391.17 | 0 |
| 11 | -2.884 | -0.4161 |  | + |  |  |  | 5 | -1397.03 | 2804.5 | 1397.74 | 0 |
| 3 | -0.1344 | -0.4166 |  |  |  |  |  | 4 | -1403.47 | 2815.2 | 1408.47 | 0 |
| 9 | -3.993 | -0.4349 |  |  |  |  |  | 4 | -1453.26 | 2914.8 | 1508.04 | 0 |
| 1 | -0.4611 | -0.4375 |  |  |  |  |  | 3 | -1461.34 | 2928.8 | 1522.08 | 0 |

And the best model is to include all of the factors.

To tested any spatial autocorrelation, we used spdep package, and we found no spatial autocorrelation (p = 0.055).

We checked the collinearity diagnostics using Variance Inflation Factors (VIF).

Table S3. Variance Inflation Factors (VIF), Tolerance, and Correlation Level for Predictor Variables in the Model

| Term | VIF | VIF  95% CI | adj. VIF | Tolerance | Tolerance 95% CI | Correlation Level |
| --- | --- | --- | --- | --- | --- | --- |
| UrbanizationRate | 1.08 | [1.01, 1.72] | 1.04 | 0.93 | [0.58, 0.99] | Low |
| PreyType | 1.07 | [1.01, 1.81] | 1.03 | 0.93 | [0.55, 0.99] | Low |
| DayN | 1.07 | [1.00, 1.88] | 1.03 | 0.94 | [0.53, 1.00] | Low |
| PredatorType | 11.11 | [8.37, 14.88] | 3.33 | 0.09 | [0.07, 0.12] | High |
| UrbanizationRate:PredatorType | 11.02 | [8.30, 14.76] | 3.32 | 0.09 | [0.07, 0.12] | High |
